# The European Network on Psychosomatic Medicine (ENPM) – history and future directions

**DOI:** 10.1186/s13030-016-0086-0

**Published:** 2017-01-26

**Authors:** Hans-Christian Deter, Kristina Orth-Gomér, Bohdan Wasilewski, Ramiro Verissimo

**Affiliations:** 1 Medical Clinic, Psychosomatic, Charité, Hindenburgdamm 30, 12200 Berlin, Germany; 2 Karolinska Institutet, Clinical Neuroscience, Stockholm, Sweden; 3 Psychosomatic Institute in Warsaw, Psychotherapy and Sexology Clinic, Postgraduate Medical University, Warszawa, Poland; 4 University Porto Faculty of Medicine, Porto, Portugal

**Keywords:** Network, Psychosomatic medicine, Research, Health care, Cooperation

## Abstract

**Background:**

Within national and international societies of psychosomatic medicine the idea has emerged of bringing together and coordinating psychosomatic, behavioural, psychological and medical actions with common interests throughout Europe as a way to increase their scientific and political influence.

**Methods:**

It was felt that there was a strong need and opportunity of a common and unifying forum for scientific exchange.

**Results:**

It was considered desirable to exchange scientific thoughts and experiences in an open minded and boundless way, among individuals and societies, between disciplines and across borders. The course of ideas and discussions within the group of European psychosomatic scientists over 12 years is presented as an effort to combine strengths and actions supporting clinical psychosomatic research and medical practice in Europe. The fields of psycho-cardiology, quality in primary care, psycho-oncology, gastrointestinal psychosomatics, C/L Psychiatry, and Psychosomatics are examples of such positive developments.

**Discussion:**

Several historic ideas are mentioned and the aims and advantages of the newly founded European Association of Psychosomatic Medicine are discussed. The advantages and virtues of a more powerful common European organisation of Psychosomatic Medicine and Psychiatric Consultation-Liaison are compared to continuing our work within the present Psychosomatic/Psychiatric and Behavioural fields.

**Conclusion:**

Psychosomatic and Behavioural Medicine have reached a strong position in Europe. There are studies in which the medical speciality is on equal terms with psychosomatic medicine representatives. There is a continuous need for scientific conferences, for teaching, and for better practice with patients. This could be coordinated by a network. Much energy and time is lost in isolated societies and countries. We want to focus our resources in scientific projects within the boundaries of a scientific network with the primary aim of developing psychosomatic scientific exchange.

## Background

In this article we describe “psychosomatic medicine” as bio-psycho-social medicine, as in G. Engel’s [1] definition, on one hand meaning a holistic dimension of medicine and on the other explaining in a scientific way differentiated bio psycho social mechanisms of etiology and the course of somatic and somatoform diseases along with possible intervention options. The importance of psychosomatic medicine has increased in both research and health care.

### In research

It is obvious that in the last century medicine has detected several mechanisms of the etiology of different diseases along with new treatments. The scope of psychosomatic medicine has grown and been spread into new dimensions. Psychosomatic scientists need all the power and support they can get from research institutions and from collaboration with each other. In this way they can maintain a high research level in this field, which has changed dramatically during the last 50 years.

### In health care

Mental disorders are highly prevalent in Europe and impose a major burden on individuals, society and the economy [2]. About twenty years ago the diagnosis of emotional disorders and psychosomatic disturbances was rare. Now, individual expectations in terms of quality of health and the phenomenon of progressive, scientific, psychosomatic understanding of diseases has increased and led to a demand for practical use of psychosomatic medicine. Acceleration in the development of significant technological advances in the field of medical science has created hope for radical improvement in life expectancy and quality of health. While life expectancy has been extended, the progress in the quality of health is unsatisfactory, mainly due to chronic, persistent emotional disorders and psychosomatic symptoms.

This new interdisciplinary setting is a challenge for practitioners – physicians, psychologists, nurses, social workers and others - and for scientists in the psychosomatic field. Many of these professionals have their own scientific societies, not only in special research fields, but also in medical specialities and sub specialities. For this reason, over the course of time different international and national societies have been formed. Compared to specialist societies like gastroenterology or psychiatry, psychosomatic or behavioural societies have a broader scope. They focus on psycho-social conditions and mechanisms according to origin and course of all somatic, somatoform and psychological diseases and want to influence their conditions by psycho-social or other interventions.

Communication between all professions in the field seems useful. The idea was born that different international and European psychosomatic/behavioural societies should be able to communicate in special research, health care, and psychosomatic training questions. This could be facilitated through special networks for scientific exchange. All medical/psychological societies involved in a special psychosomatic issues should be able to cooperate to maximize their strengths (and their ability to write research proposals for grants) in the competition with genetic, biochemical, pharmaceutical, cardiologic and other powerful research groups. This article describes an attempt to increase communication between the professions involved in psychosomatic medicine. Beginning with the history of ECPR, following with a description of the ENPM aims and development, the combining of ENPM and EACLPP and the limited success of this cooperation (see below), future directions of the aims and ideas of ENPM are outlined at the end of this paper.

### History of ENPM

The European Network on Psychosomatic Medicine was founded during the joint 25^th^ ECPR- EACLPP meeting held in Berlin 2004 as a forum for 21 delegates of many psychosomatic/behavioural/psychiatric/internists national societies to present their work*.

*Members of the ENPM initiative 2004/2005 were: Gunta Ancane (LV), Margarita Beresnavaite (LIT), Antonio Barbosa (PT), Hans-Christian Deter (GER), Dan Dumitrascu (ROM), Kristina Dropowa (POL), Christian Facekas (AU), Giovanni Fava (IT), Per Fink (DK), Maria Kopp┼(HUN); Ulrik Malt (NOR), Gabriele Moser (AU), Kristina Orth-Gomér (SE), Carl Scheidt (GER), Gerhard Schüssler (AU), Tatjana Sivik (SE), Wolfgang Söllner (GER), Törres Theorell(SE), Ramiro Verissimo (PT), Ad Vingerhoets (NL), Bohdan Wasilewski (PL)

An important task was to promote scientific exchange and collaboration between members of different societies and medical fields. One impressive example of such cooperation was the “Task Force for European Guidelines in prevention of cardiovascular disease”. This was concerned with the formulation of rules and recommendations on how to prevent recurrences in heart patients. The group consisted of representatives of several different societies - Cardiology, Atherosclerosis, Diabetes, Hypertension, Behavioural Medicine, Family Medicine etc. The psychosomatic contribution of the organized work group for these Guidelines was truly international and interdisciplinary. Another form of activity, centered mainly in the area of Eastern Europe, was the activity appointed by ENPM in 1994 - European Training Centre (ETC) on psychosomatic medicine, acting in Warsaw. In cooperation with the Polish Psychosomatic Society and Psychosomatic Institute, ETC has implemented educational projects in cooperation with the Polish Ministry of Labour and Social Policy - a semester program of postgraduate training for more than 600 social workers. They were trained to recognize emotional and psychosomatic disorders and to participate in comprehensive treatment.

**Fig. 1:**
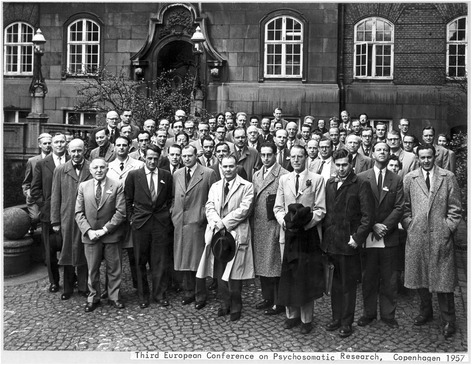
Participants of the 3^rd^ ECPR in Copenhagen 1957. 49 men and 5 women; 1^st^ row from left: Johannes Groen, Amsterdam, Dennis Leigh, London; 4th.from left: G. S. Philipopoulos, Athens; 5th.from left F. Antonelli, Roma, 7^th^ from left Lennart Levi, Stockholm; 3^rd^ row 1^st^ from right Arthur Jores, Hamburg, 4^th^ row behind G.S Philipopoulos right: Finn Joergenson, Copenhagen; 3^rd^ row, 5th from left Yasutaro Satake (1884–1959) who used to be 8^th^ President of Tohoku University

The forerunner and important model for the ENPM was the European Conference on Psychosomatic Research (ECPR); the first of which took place in London in 1955. These conferences brought together individuals from European countries interested in psychosomatics [3]. The first three Conferences took place annually; in London, Amsterdam, and Copenhagen (Fig. 1). Then there were two bi-annual conferences in Hamburg and Madrid; after which there were conferences every three years, until 1970, with venues in Athens, Rome and Knokke in Belgium (Table 1). Elected four years earlier among the community of European researchers, a well-known European researcher was the president and organizer of each conference. Interestingly, 60 years later we recognize distinguished psychosomatic scientists who were among these successive organizers of the ECPR’s meetings o.g. Johannes J. Groen, Archibald Denis Leigh, Lennart Levi. A formal society did not seem necessary in those days, when communication was a very individualized process. The main goal of these meetings was to modernize the psychosomatic medicine focus from literature and philosophy into comprehensive research oriented toward acquiring better and sounder knowledge in psychosomatics. It seemed necessary by then to come forward with evidence-based findings obtained through experimental research and studies on the psychosomatic underpinnings of different diseases. Of relevance to this matter were the London group, D. Leigh, psychiatrists from Madrid, J. J. López Ibor and Italy, Ferrucio Antonelli, as well as internists from Amsterdam and Hamburg, J. Groen, Henk Pelser, Arthur Jores. From the 1950′s, the group was able to present, discuss and promote their own studies in the scientific journals “Psychotherapy and Psychosomatics” (1953) and “Journal of Psychosomatic Research” (1957).

**Table 1:** Presidents and locations of the European Conferences of Psychosomatic Research (ECPR)

• D. Leigh (London 1955)
• J.J. Groen (Amsterdam 1956)^a^
• V. Lunn (Copenhagen 1957)
• A. Jores (Hamburg 1959)
• J. Rof Carballa & J.J. Lopéz-Ibor (Madrid 1961)
• G.S. Philippopoulos (Athens 1964)
• F. Antonelli (Rome 1967)
• R. Pierloot (Knokke 1970)
• E. Ringel (Vienna 1972)
• C. Aitken (Edinburgh 1974)
• W. Bräutigam (Heidelberg 1976)
• F. Askevold (Bodø 1978)
• G. Koptagel-Ilal (Istanbul 1980)^a^
• H. Pelser (Nordwijkerhout 1982)
• H. Wolff (London 1984)
• G. Christodolou (Athens 1986)^a^
• W. Schüffel (Marburg 1988)^a^
• P. Tienari (Helsinki 1990)
• (1992 in Dubrovnik cancelled due to Bosnian war)
• M. van Moffaert (Gent 1994)
• M. Bourgeois (Bordeaux 1996)
• F. Creed (Manchester 1998)^a^ ^b^ ^c^, founding of EACLPP with common biannual and separated biannual meetings
• U.F. Malt (Oslo 2000)^b^
• G. Cardoso & A. Barbosa (Lisbon 2002)^a^
• H.C. Deter (Berlin 2004)^a^ ^c^
• M. Talcic (Cavtat 2006)
• A. Lobo (Zaragossa 2008)^b^
• G. Schüssler (Innsbruck 2010)^b^
• P. Fink (Aarhus 2012)^a^ ^b^ ^c^, founding EAPM with annual meetings
• D. Dumitrascu (Sibiu 2014)^a^ ^c^

Since 1986: ^a^ICPM member ^b^EACLPP member ^c^APS member (limited information before 1986)

Presidents of the International College of Psychosomatic Medicine, see paper of J. Streltzer in this series 2016)

Presidents of the International Society of Behavioral Medicine, see paper of K. Orth-Gomér & N. Schneiderman in this series (2016)

At the time ENPM was founded five other societies were already involved in the “Psychosomatic field”:

1. *The American Psychosomatic Society (APS; see also the paper by Herrmann-Lingen and Drossman. in this volume, 2016)*
  With a tradition going back to the 1930s, founded in 1942 by a group of scientists: Edward Weiss, Helen Flundars Dunbar, Walter B. Cannon, Eric Lindemann, Harold G. Wolf et al., this society was mainly oriented toward psychobiology into the detection of psycho-social mechanisms involved in somatic diseases. In later years it has become increasingly difficult for APS to host research, health care, and clinical practice under the main scope of this society. Consequently, APS has renamed itself in the last years as “APS, dedicated to the integration of biological, psychological and social factors in medicine”. The *journal of the APS* “Psychosomatic Medicine”, was founded 1939, and now carries the subtitle: “Journal of Bio-behavioural Medicine”.
2. *The International College of Psychosomatic Medicine (ICPM; see also the paper by J. Streltzer in this volume, 2016).*
  This society was founded by scientists from North America: Eric Wittkower, Morton Reiser, Zbigniew J. Lipowski and Adam Krakowski, South America: Maurice Knobel, Roberto Kertész, and Europe: Herman Musaph, Johannes Groen and others in 1970, and included representatives from Asia (Yujiro Ikemi), Africa (Henry Collomb) and Europe (Jan Bastians, Jules Angst, Thure v. Uexküll). It used to have a biannual meeting that alternated with the European Conference on Psychosomatic Research. This society was more focused on the medical field as a whole and on a holistic perspective of medical practice. The stimulation of better psychosomatic clinical care in the broad medical field was equally as important as the level of research. George Engel, from Rochester, was a mentor and keystone to this thinking [4]. This society also publishes in the *ICPM journals* “Psychotherapy and Psychosomatics”, the “Journal of Psychosomatic Research”, and “General Hospital Psychiatry”.
  Following the ideas of ICPM was the founding of an Asian College of Psychosomatic Medicine by Internal Medicine physicians from Japan (1984). They had founded their own Japanese Society 1959 [5] and were also interested in the integrative perspective of Psychosomatics in the whole field of medicine (see the paper of Y. Nakai and M. Murakami in this volume, 2016). Of the many societies (from Spain, Italy, etc.), the German College of Psychosomatic Medicine was one of the first national European societies, founded in 1974, and had ideas and activities closely related to those of the ICPM (see the paper by S. Zipfel et al. in this volume, 2016).
3. *The Academy of Psychosomatic Medicine (APM*; Psychiatrists providing collaborative care bridging the gap between physical and mental health) founded by psychiatrists interested in consultation-liaison (C-L) psychiatry and psychosomatic medicine 1953 (W. Dorfman, Z. I. Lipowski [6, 7] and others), APM maintained that psychosomatic medicine was very close to the clinical perspective and practice of psychiatrists working in the field of consultation-liaison activities in general hospitals. This overlaps the EACLPP conception (see below); but its tradition goes back to the 1950′s. The APM (1200 members, 900 congress participants) is a member of the American Psychiatric Association and has had its main *publication forum* in the journal “Psychosomatics” since 1960.
4. *The International Society of Behavioural Medicine (ISBM; see also the paper by Orth-Gomer & Schneiderman in this volume, 2016).*
  Founded in 1990, by five national societies of behavioural medicine (Stephen M. Weiss, Irmela Florin, Kristina Orth-Gomér et al.), ISBM defined “behavioral medicine as the interdisciplinary field concerned with the development and integration of biomedical, behavioural, psychosocial, and sociocultural science, knowledge and techniques relevant to the understanding of health and illness, and the application of this knowledge to disease prevention, diagnosis, treatment, rehabilitation and health promotion” [8]. It focused on all-important behavioural, psychosocial and biological risk factors and had as its goal the detection of behavioural, psychosocial risk factors besides “biological mechanisms” in the social environment. With lesser emphasis on individual psychosomatic processes and more emphasis on public health, it was founded by both physicians and psychologists [9, 10] and focuses mainly on sound empirical research. The integration of behavioural medicine with other scientific fields would lead to better and more successful research. ISBM is an umbrella organization and has 26 national or regional societies (representing many thousand individual members) over the whole world. Formerly, psychosomatic societies were based on individual membership. The *journal of this society* “International Journal of Behavioural Medicine” started in 1994.
5. *The European Association of Consultation Liaison Psychiatry and Psychosomatics (EACLPP).*
  Founded in 1998, it was an attempt to solve the problem, as some researchers saw it, of the loose structure underlying the organization of the European Conferences on Psychosomatic Research. The founding members meant to provide a means to work together more intensively within a society of their own. The starting point of the EACLPP was the 1987 decision of some consultation-liaison (C-L) psychiatrists in Europe to develop a closer collaboration to stimulate the development of the C-L field [11]. Following this initiative, the European Consultation-Liaison Workgroup for general hospital psychiatry and psychosomatics (ECLW) was established. The group consisted of psychiatrists and psychologists working with patients referred to psychiatric/psychosomatic departments. These scientists designed a huge project, the ECLW study [12], sponsored by the European Union. The study included 226 consultants from 56 psychiatric C-L services in 11 countries. The ECLW study required that a network of researchers and clinicians across Europe be established [13]. When the ECLW study ended, the EACLPP was established as a formal organization of the ECLW network. These researchers were mainly focused on “Consultation-Liaison diagnosis and care in a general hospital setting as applied by psychiatry and psychosomatic physicians [14]. Additionally the C-L section of the European Association of Psychiatry organizes symposia and education in psychosomatic medicine, with emphasis on psychiatric aspects. The “Journal of Psychosomatic Research” became the *scientific platform of EACLPP*.

There is little distinct difference in content of the various societies, they all try to integrate body and mind, but there are clear differences in methods, aims, objectives, and health care practice.

6. *Other societies in the “psychosomatic field”*
  Societies of psychophysiology, psycho neuro-immunology, health psychology etc. were also interested in this approach to the medical area, while focusing on epidemiology, physiology, biochemistry and interventions for some special patient groups.
  Special interest groups and organizations related to specific disorders or treatments also had their own societies: e.g. European Association of Palliative Care, European Work Group on Transplantation Psychology and Psychiatry, International Society in Dermatology, Psychiatry and Psychosomatics, International Society of Psychosomatic Obstetrics and Gynaecology with national branches, European Association of Communication and Health.
  Psychotherapeutic societies and psychotherapeutic research in the psychosomatic field
    Also important are the developments that occurred in the psychotherapeutic scene、 which influenced psychosomatic medicine; namely the founding of the International and German Psychoanalytic Association (1910/1926) and the German Society of Psychotherapy (1928); which influenced the founding of APS. The International Federation of Psychotherapy, the Society of Psychotherapeutic Research and the different national societies of Behavioural Therapy also left their traces on the psychosocial dimension of Psychosomatic Medicine interventions today, e.g. the European Association for Behavioral and Cognitive Therapies (EABCT). It is an association that brings together 53 individual associations from 39 different countries. Each association is committed to empirically based principles and the practice of behavioral and cognitive therapy approaches in the health, social, education and related fields. They include studies on CBT in somatic diseases and of patients with somatic symptoms. Additionally, Germany has developed a medical specialty, the “German Society for Psychosomatic Medicine and Psychotherapy”, which was founded in 1990 ([15], see Zipfel et al. in this volume).

While two of the five international psychosomatic societies mentioned above were founded in the United States, the others had a European traditional background. The different developments of these international psychosomatic societies probably are an expression of the conceptual and psychotherapeutic (psychodynamic, psychiatric or behavioural) way of thinking of their members (Table 2). However, in the middle of the first decade of 2000, the time had come for a common interdisciplinary perspective and practice, free of ideological and professional “blind spots”.

**Table 2:** The old world meets the new. Origins of psychosomatic medicine: concepts, scientific operationalisation and health care implementation in different psychosomatic communities

Societies	Membership special profile^b^	Research	Health care
European Conference on Psychosomatic Research ECPR (inaugurated 1955)	European; interested physicians and psychologists on the biannual conferences, 450 participants, 250 posters; no society, no members. At the conferences one business meeting of ECPR participants	Research on psychosomatic diseases in a bio-psycho-social way and applying this knowledge into clinical practice; focused on clinical psychosomatic research, mechanism and interventions	Health care issues in the whole field of medicine
International College of Psychosomatic Medicine ICPM (Inaugurated in 1970)	International; 120 individual members from 30 different countries around the world, professionals (physicians, psychologists, nurses etc.) in health care. Bi-annual meetings (600–1000 participants; 200 posters), president, board, advisory board, 3 committees^c^ Implementation of psychosomatic knowledge in clinical practice; focusing on doctor patient relationship and emotional aspects in Psychosomatic Medicine	Common clinical and philosophical questions of the whole clinical field, interventions	Practical issues of the whole field of medicine, many specialities, like general practitioner, internal medicine, gyneacology.
International Society of Behavioural Medicine ISBM (Inaugurated 1990)	International Federation of 26 regional member societies around the world (14 European); about 20% physicians/80% psychologists and others. Bi-annual meetings (650–800 participants; 400 posters), president, executive committee, governing council, 9 other committees, 4 special interest groups^c^; newsletter. On one hand epidemiological, public health and on the other hand neuro-biological aspects of empirically found associations. Identification of four important phases: 1. Identification of the health problem. 2. Re-evaluation. 3. New methods to manage the problem. 4. Training of skills to maintain change.	Mainly focused on behavioural aspects of medicine; emphasis on cognitive behavioural intervention and prevention; recognition of behavioural mechanisms in public health. Health care politics.	Focusing on behavioural aspects in medicine, imple-mentation in primary care and other specialties with scientific evaluation.
European Association of Consultation Liaison Psychiatry and Psychosomatics EACLPP (Inaugurated 1998)	About 100 individual members, mostly psychiatrists. Annual meetings (200 participants; 100 posters), president, board, working and special interests groups. Research in the field of Consultation Liaison psychiatry and psychosomatics with integration in hospital and clinical practice of psychiatry and the field of medicine	Clinical psychiatric/psychosomatic research, inter-ventions;development of the Care Complexity Predic-tion Instrument (COMPRI) and INTERMED as spin-off of the ECLW study [29]	C/L Psychiatry and Psychosomatics, Integrated care
European Association of Psychosomatic Medicine^a^ EAPM (Inaugurated 2012)	About 120 individual members, psychiatrists, psychosomatic specialty, psychologists. 10 European member societies. Annual meetings (250–400 participants; 150 posters), president, board, 1 working and 13 special interests groups^c^. Research in the field of Consultation Liaison psychiatry and psychosomatics, integration in the whole field of psychosomatic medicine, hospital and clinical practice.	Clinical psychosomatic and psychiatric research, interventions	Psychosomatic Medicine, C/L Psychiatry and Psychosomatics, Integrated care
American Psychosomatic Society APS (Inaugurated 1942)	North American society; about 1300 individual members, psychologists, physicians, few specialties; with an international branch (about 12% from Europe); annual meetings (500 participants; 800 posters), president, board, 6 committees, 5 special interest groups^c^; newsletter (twice a year) ;	Goals: Scientific excellence, clinical relevance; mainly focused on clinical psychosomatic research: mechanism; intervention studies (RCT)	Interested in special psycho-somatic fields: p.e. cardiologic, gastrointestinal, pain etc.

^a^EAPM was founded in response to reorientation of the European psychosomatic development, to combine ideas of ECPR and EACLPP

^b^Member-, participant- and poster-numbers of this table are information that the authors obtained from conferences, newsletters, websites or in personal communication within the last few years. They are not fixed on a special time point and roughly estimated. For exact information within a special timeline, please contact the secretaries of the individual societies

^c^Topics of the individual committees, special interest and working groups are shown on the individual society website: www.icpm.org (3); www.isbm.info (13); www.eapm.eu.com (14); www.psychosomatic.org (11); www.apm.org (26)

### Ideas, aims and progress of the ENPM

The “European Network of Psychosomatic Medicine” (ENPM), dedicated to the integration of psychological, social and biological factors in health care”, was established, after a first meeting in 2004, by colleagues from European countries participating in the European meeting held in Berlin in 2005 (July 8/9). It was open to all national and international psychosomatic societies and colleges, ECPR-organizers, EACLPP, ICPM, ISBM and others.

This network was meant to be open to all European and international scientists and clinicians, as well as psychosomatic, psychiatric and behavioural societies also interested and working in this field. The founding members attending this meeting were, in one way or another, also involved with the Psychosomatic Societies from Sweden, Poland, Latvia, Hungary, Romania, Portugal, Austria and Germany; all other European and International Societies were then invited to join the European Network for Psychosomatic Medicine (ENPM).

Communication among scientists was anchored in an ENPM Homepage (http://www.enpm.eu), which included hypertext links to the web-pages of all European Psychosomatic societies. The management of the Network website as well as the commitment of proposing a logo was assigned to R. Verissimo, from Porto University, Portugal. RV and HCD conducted the developmental work on computer tools and soft ware, which have enabled us to implement the ideas of free and integrative scientific exchange of ideas, concepts, thoughts, results, and conclusions. An important aim was not to engage the members in any unnecessary administrative tasks. A new model of free scientific integration that will directly benefit the quality of our scientific work and personal competence is practised. The model of psychosomatic medicine did not differ from those presented by other psychosomatic societies or associations, but the focus on communication over society borders was new.

The German College of Psychosomatic Medicine assumed in turn to host an internet discussion forum on their homepage involving all members of the ENPM, and C. Scheidt was appointed as the first manager of this forum.

Perspectives of collaboration in education and research [16]:

- Recognition, discussion, and harmonisation of students and postgraduate training in psychosomatic medicine was assumed to be one of the outmost importance tasks for ENPM
- Promoting psychosomatic oriented health care in a European perspective, in general practice, and other specialties (dermatology, gynaecology, neurology etc.), was another important task also considered.
- Psychotherapeutic training for medical doctors and psychologists, and their integration within the health care system (in private practice and at an inpatient level) was a topic of interest.

The need for common European actions in the field of Psychosomatic Medicine

- Psychosomatic medicine in Europe must deal with similar problems and themes, such as the relation between theoretical findings from different fields: biological, on one hand, from basic sciences, and progress in good clinical practice on the other.
- This means good bio psychosocial primary care, family and internal medicine and detection of psychosomatic mechanisms implicated in different chronic diseases.
- As we gain a better understanding of the mechanisms involved in these complex diseases, especially on the psychosocial influences, we should also develop strategies to promote this knowledge in each and every country, thus allowing its implementation into their medical practice.

Research in psychosomatic medicine is often conducted in collaboration with somatic colleagues, but to demonstrate psychosomatic interactions involved in some diseases we need good empirical background data in all medical domains. We have to provide evidence that special psychosomatic strategies of treatment are better for dealing with biological, psychological, and social aspects involved in these complex diseases; and we have to demonstrate, through randomized clinical trials, that the efficacy of these treatments is, at least, comparable to other commonly used treatments. Only in this way will it be possible to bring psychosomatic experiences and knowledge into a level of widely accepted national and international guidelines for these complex diseases. This seems to be a program that can be independently adopted by many psychosomatic research centres. The interdisciplinary communication and integration of important ongoing studies that the European Network on Psychosomatic Medicine intended to foster combined ideas and actions and made the acquired psychosomatic knowledge available to the health care systems across Europe.

Aims of the network

- Bring together all psychosomatic and behavioural societies in the psychosomatic field
- Coordinate European research activities sponsored by the European Union
- Coordinate European exchange programs for students, postgraduates and other research fellows
- Discuss actual important psychosomatic/behavioral/CL questions
- Give support for developing psychosomatic national societies

Actions, that promote the efficacy the integration of the ENPM:

- Proposals for EU grants, to promote the scientific process of co-working in Europe and the eastern countries
- Make common studies with EU-funding
- Inform about and combine common interests in different national psychosomatic/behavioural/CL-psychiatry societies

Discussions at the homepage: http://www.enpm.eu

- Links and contacts to all national and international Psychosomatic/Behavioural societies in Europe
- Open discussion platform for several questions in the psychosomatic field
- ENPM coordinators in all European countries, who give support for the ENPM

Topics for action

- Psychosomatic training and diploma in Europe
  Coordinator: G. Schüssler, Innsbruck, Austria
- Psychosomatic/behavioural interventions in Coronary heart disease in Europe
  Coordinator: K. Orth-Gomér, Stockholm, Sweden, European Guidelines in Cardiovascular Prevention in Clinical Practice.
- Psychosomatic/behavioural interventions in ulcerative colitis and Crohn’s disease in Europe. Coordinator: G. Moser, Vienna, Austria, European evidence-based consensus on the diagnosis and management of ulcerative colitis [17]
- European exchange programs for students, postgraduates and other research fellows
  Coordinator: Dan Dumitrascu, Cluj, Romania.
- Psychosomatic basic care in Europe
  Coordinators: B. Wasilewski, Warsaw, Poland; H.-C. Deter, Berlin, Germany. A program implemented in 1995 with the participation of the ETC, Psychosomatic Institute in Warsaw and the Polish Balint Association is a training program for Ukrainian doctors and psychologists in the field of doctor-patient communication and psychosomatic approach in medical and psychological practice (B. [19]). Under this program, implemented in cooperation from the Ukrainian side by Bukovinian State Medical University in Chernivtsi and the Association of Psychotherapists and Psychoanalysts of Ukraine, several hundred Ukrainian doctors and psychologists participated in training.
  An initiative to obtain EU-funding for research for “Communication in doctor-patient relationship” was initiated.

Meetings which took place with ENPM participants, presentations, symposia, work-shops and business meetings between 2004 and 2015 were held at European, national and international Psychosomatic Conferences in Cavtat, Croatia, 2006; Zaragoza, Spain, 2008; Innsbruck, Austria, 2010; Aarhus, Denmark, 2012 (European Conferences on Psychosomatic Research (ECPR); and Sibiu, Romania, 2014 (EAPM). National meetings of the German College of Psychosomatic Medicine were held in Nuremberg, Freiburg, Mainz, Essen, Munich, Heidelberg, Berlin and of the Polish Psychosomatic society (English language in international sessions).

In 2008 a broad vision was presented. It was general and wide enough to include the aims of the ENPM and other psychosomatic/behavioural societies in Europe for the next 20 years (Table 3). The development of the ENPM was a practical organization process to frame those different and overwhelming aims. It seemed unrealistic and out of reach to manage those aims without a proper structure of its own society.

**Table 3:** Visions in psychosomatic medicine for the year 2030 [18]

Research - Basics
• The basic sciences have all bio-psycho-social relations in a neuroscience perspective examined and origins of infections, immunity, CHD, carcinoma, asthma and other diseases shown.
Research – Health care
• National and European randomised multi centre studies (RCT’s) with psycho-social interventions are done with all important chronic diseases
• Their results are integrated in all international/European guidelines according to psycho-social diagnostic and therapeutic aspects
Training for specialists in primary care, internal medicine, psychiatry and others
• In Europe and all other countries there is a psychosomatic diploma for physicians
• They have the capability to diagnose psychosomatic diseases and apply different therapeutic techniques, which are necessary for psychosomatic health care
Psychosomatic basic care and specialized care
• in C/L Psychiatry and Psychosomatics in all European hospitals
• Standardized out-patient psychosomatic health care in all specialties
Prevention
• Good and successful strategies of disease prevention and health care

### Further steps of the ENPM

In Innsbruck 2010, the ENPM decided to found a new society, the European Federation of Psychosomatic Medicine, with a president, treasurer, and secretary, to foster interaction between individual members and different European Psychosomatic societies that would include the above-mentioned basics. After the founding meeting in Innsbruck, the idea came up of merging the ENPM - an informal network of scientists and friends - with the much more structured society EACLPP. This was done after many, partly intense discussions, among colleagues and board members of ENPM and EACLPP at the meetings in Aarhus 2012 and Cambridge 2013. The election of a European Association of Psychosomatic Medicine board took place. Since then three annual EAPM conferences (Sibiu, Nuremberg, Lulea) have been organized.

### Commentary

There are many national and international scientific societies active within the psychosomatic field (Table 2). As compared to primary care, gastroenterology [20] or cardiology, where one powerful society is active (e.g. the European Society of Cardiology, with more than 20,000 participants at the annual meetings), the field of psychosomatic/behavioural medicine is broader. It is in contact with all societies that represent the different medical disciplines and sub-disciplines [21]. The psychosomatic interest area (psychosomatic medicine, behavioural medicine) is also spread throughout many different scientific groups oriented or devoted to special aspects: psychosocial care/intervention, primary care or even special sub-disciplines like medical/clinical communication, psychophysiology, psycho-neuro-immunology, psychosomatic public health, health psychology, and others. All these scientists are innovative and are working in important fields of psychosomatics, but mostly without cooperation with other members of different psychosomatic sub-disciplines. The scientific journals of each society give important and new information for psychosomatic scientists about progress and new events in a special field. But, it seems necessary to intensify and combine the activities of these diverse societies involved in psychosomatic medicine. In fact this is a very diverse field. The debates on its value for clinical aspects of diagnosis and treatment are so controversial that it was necessary to promote more intense collaboration and discuss the different scientific questions raised in many groups, but also within a European Network on Psychosomatic Medicine.

This idea may be in conflict with the engagement of the individual professional groups, involving different disciplines. The structure of each group is crucial for the aims, ideas, and self-confidence of the individual members of these groups. But the situation now may be good for the field of psychosomatic medicine and its researchers. The example of the 3^rd^ Task force of European Guidelines on cardiovascular disease prevention, where eight societies worked together for scientifically based high level recommendations for clinical practice, is instructive in that it encouraged us to organize a communication platform for psychosomatic and behavioural medicine in Europe [10, 22].

### European Network on Psychosomatic Medicine (ENPM) and the attempt to merge it with EACLPP

The question for the newly founded society EAPM was which way to go. This was not only a network activity for European researchers on the same level, but included now also a president, vice president, board, the EAPM members, and the associated societies of EAPM. What should be the targets and challenges of the new society in the area of European psychosomatic medicine (Table 2)?

Firstly, a clear definition:

1. Psychosomatic medicine in research and health care may imply:
  - Psychological and social aspects of etiology and course of somatic diseases. This includes personality and behavioural aspects e.g. classic conditioning, operant conditioning: prevalence, impact on course / outcome. It also includes psychosocial interventions.
  - Psychological and social aspects of etiology and course of somatoform/functional disorders and other psychological syndromes with somatic symptoms. (Including personality): prevalence, impact on course / outcome. It also includes psychosocial interventions.
  - Psychiatric aspects of somatic, somatoform diseases and other psychological syndromes with somatic symptoms: prevalence, impact on course / outcome. It also includes psychological interventions. There is a discussion if psychosomatic medicine includes psychotic or only non-psychotic disorders like anxiety and depression.
  - Psycho-neuro-pathophysiology, -endocrinology, –immunology of a, b and c
  - Population based studies on prevalence and incidence
2. In a holistic perspective the following important points have to be added:
  f. understanding and improvement of communication and interaction between patient and physician or other care givers,
  g. critical view on rationale, structure and development of health care systems in a society and
  h. examination of health care systems under bio-psycho-social needs of patients and doctors

In psychosomatic practice, a tendency to focus on special aspects of clinical care, e.g. C/L psychiatry, psychotherapeutic medicine applied by physicians, or behavioural therapy in medicine, can be identified. Such limitations are not necessary and will not be widely accepted by others (e.g. ICPM, ISBM), they do not present the whole field. For the challenges of psychosomatic medicine, mentioned above, it seems important to focus on crucial points.

Our goal was to foster international and European psychosomatic/behavioural societies. How should they communicate and cooperate in special research, health care and psychosomatic training questions? We saw the importance of establishing networks to combine the strengths of all societies working in the psychosomatic field.

1. There seems to be a high need to discuss strategies for psychosomatic research in the future in special disease networks. A small society like EAPM – focused on clinical research and care - does not fulfill those requirements and cannot give sufficient support for a big study like the EU funded Consultation-Liaison study [13] or the Female Coronary Risk study [8]. We think this society is too small and, the perspective too narrow to organize, within scientific groups of somatic medicine, a big study or work together with large groups in a European Guidelines committee [22].
2. The different challenges related to the level of health care and services are a second task. One individual society should focus on all care levels: e.g. GP-, clinical specialty- and CL psychiatric/psychosomatic service level, which have different clinical needs and scientific foci. Individual training and learning by doing through the responsible GP’s or physicians in the specialties or support from psychosomatic specialists are two kinds of psychosomatic care: Responsible physicians in the whole clinical field as well as psychiatrist or psychologists working in general hospitals have to select and pursue different tasks.
3. A third point was the challenge to increase psychosomatic knowledge and skills in different professionals working in the field of psychosomatic medicine, e. g specialists in internal medicine, psychiatrists, psychologists, nurses, and social workers. They have different needs. It is impossible for EAPM to sufficiently influence the professional standards in one region, one country, or in the whole of Europe.

What happens with the aims of the former ENPM after the decision has been made to cooperate in one single society (Table 4)? EAPM started a really good process in developing by-laws and an exemplary administration, having now at the annual meetings delegates from 23 European countries, integrating ten national societies of C-L-psychiary and psychosomatics (5) and Psychosomatic Medicine (5) as members; which was one of the goals in ENPM. EAPM could cooperate in the conferences 2014, 2015 and 2016 in common satellite symposia with ICPM or ISBM.

**Table 4:** Aims, discussions and actions developed by ENPM only partly realized in EAPM

ENPM aims	EAPM, June 2016
Aims	
• Bring together all psychosomatic and behavioural societies in the Psychosomatic field	• 4 psychosomatic societies • 1 primary care society • 5 consultation/liaison/psychiatric societies 120 individual members
• Coordinate European research activities sponsored by the European Union and influence decisions of national and European health care- and research politicians	• none
• Coordinate European exchange programs for students, postgraduates and other research fellows	• Partly; 2015 Academy for Psychosomatic Medicine was founded
• Discuss actual important psychosomatic questions	• few, many are missing
• Give support for developing psychosomatic national societies	• For the Romanian society only
Discussions at the homepage: http://www.enpm.eu	http://www.eapm.eu.com
• Links and contacts to all national and international Psychosomatic/Behavioural societies in Europe	• Yes, but very few to member societies
• Discussion platform for several questions in the psychosomatic field	• Open discussion platform not accepted, very few in the membership only section of the EAPM website
• ENPM coordinators and discussion partners at the platform	• 23 delegates, open discussion platform not accepted
Actions, that promote the efficacy and the integration:	
• Proposals for Marie Curie grant of the EU, to promote the scientific process of co working in Europe and the eastern countries	• Not until now
• Common studies with EU-funding	• No proposal until now
• Combine common interests between national psychosomatic societies	• No activity to combine common interests in Psychosomatic Medicine
Proposed first steps for discussion and actions	
• Psychosomatic training and diploma in Europe	• Partly, EAPM satellite symposium with ISBM and ICPM • Academy for Psychosomatic Medicine • No attempt for organization a psychosomatic diploma • No attempt for e-learning activities within psychosomatic/behavioural societies
• Psychosomatic/Behavioural interventions in Coronary heart disease in Europe	• This working group is active
• European exchange programs for students, postgraduates and other research fellows	• ERASMUS program is still working
• Psychosomatic basic care in Europe	• An new attempt for basic care has focused on: pain and somatoform disorders in primary care

The founding of EAPM stimulated new ideas in the former EACLPP (to be more integrative, more interdisciplinary, and multi-professional), but the three main targets of the former ENPM (see above) could not be activated and stimulated. Additionally the communication among scientists (4) was anchored in the ENPM-homepage, which included links to the web pages of all European Psychosomatic societies. But the cooperation with other somatic medical societies, e.g. European Guidelines in different somatic diseases (5), giving support for psychosomatics in primary care (6), developing a psychosomatic diploma in European countries (7), or support European exchange programs for students, postgraduates and other research fellows (8) were not intended. ENPM-perspectives of collaboration in communication, research, care, and education and the results within the EAPM after four years of co-working are described in Table 4.

However we have to accept that the EAPM is a standard society with common ways of thinking and acting, which was unfortunately impossible to discuss and address in an adequate way.

- Research: There are several successful national research projects, but there was no interest in international research initiatives, not on an EU- level, not on an NIH-level, or not even on a low level towards a common European proposal for funding in the clinical somatic field. Until now there has been no attempt, whatsoever, in any psychosomatic/behavioural society, to achieve common European Guidelines (perhaps a “transplantation group” or a “somatoform disorder in primary care group” will develop). The questions cannot be answered as to who will provide for qualified research - within or outside the society- or as what kind of support is needed. Who is in the best position to get high impact (Impact Factors) and obtain grant-money for the psychosomatic field?
- Care: There was less interest in involving specialists in internal medicine, neurology, dermatology, and gynaecology in the society or working together with their specialist societies, although within those specialties the most psychosomatic cases are diagnosed and treated. Most EAPM members had psychiatric training and their main interest was health care on a consultation/liaison level with a special interest in somatoform disorders. Additionally, physicians with German psychosomatic specialty training have become members, so the society which should prevent further atomization of medicine and support the psychosomatic approach as an integral part of each medical practice, rather leaves this activity to the specialists. One question stood out already at the beginning of APS, ECPR and ICPM: Combining basic psychosomatic forthcomings in health care with a high scientific standard: Practitioners were interested in clinical aspects, but their symposium submitted to the latest psychosomatic conference was not accepted. It seems necessary to understand special psychological and biological conditions within the clinical practice domain, which cannot be easily grasped by conventional research concepts. The society has to decide how much clinical practice description is acceptable at psychosomatic conferences and which methods used in psychosomatic research are effective. The time has come to look for new answers to deal with present and future conditions.
- Training programs: Similar to the ideas and work of the American Academy of Psychosomatic Medicine, EAPM started an academy in 2015 aiming to teach psychosomatic techniques in countries without resources; which was one of the ENPM tasks (see above). There have already been several C/L psychiatry-courses e.g. in Berlin- and Manchester [23], with the focus on psychiatry and somatic disease, but with large differences across European countries [24]. Previous discussions have focused on a European diploma in Psychosomatic Medicine obtained through special training courses [25] or through an e-learning program in behavioural medicine and psychosomatics [26]. Coordination was lacking, as was discussion and communication with other international psychosomatic organizations working in this field.
- Common discussion forum at the website for all European scientists with and without EAPM membership. At the EAPM-website there are few links to national and international psychosomatic societies working in Europe, and the discussion platform, which is not very often used, is located in the membership only section. The special interest groups/working groups give only information about their activities in the membership only section, but there is no discussion with important European scientists in this field. Thus, our ideas about free and intense scientific exchange have not been implemented.
- Organizational issues: In the long run, each society, working by itself, can only achieve relative success. This was one of the arguments for unifying and bringing together collaboration through communication and integration in the way meant by ENPM.

### ENPM summary and future directions

We want to propose target areas for EAPM activity, according to our earlier ENPM ideas. Different aspects require different solutions. One intervention that works for one target group may not work for another group. One reason is that at least three different professions are involved in psychosomatic care and research. They are psychiatrists, psychologists, and specialists in internal medicine or other specialties.

Researchers have different interests and agendas: E.g.some research has a bias towards psychological-psychotherapeutic and psycho-physiological aspects of diseases, others focus their research primarily on co-morbid mental and somatic diseases and how to- intervene, including drug treatment [27]. This implies that some will want to attend “somatic” and psycho-physiological meetings, while others may tend to attend psychiatric meetings. It is by no means obvious that a network at the beginning will include all those aspects, so these suggestions must be seen and developed much more specifically and focused. “One size fits all” will not work, but it seems important that a first step focus on co-working between groups and overcoming barriers between individuals and organizations.

In our experience, this is not an easy way. After an intense discussion of these thoughts, the EAPM board minimized or declined (March 2016) to build an ENPM-discussion forum at the EAPM-website (free part) with separate platforms for interested scientists in working and special interest groups and with links to European national and international psychosomatic societies or to elect one or two EAPM delegates/board members who would be responsible for continuous cooperation with the different psychosomatic and behavioural scientific groups/societies in Europe.

Perhaps some of the EAPM members want to communicate with others, but it may be questioned to what extent they can succeed. The main difference between ENPM and EAPM remains the society structure, which was focused on the their own conditions/by laws and their own membership and which tried to built a closed shop (not only on the web site). A specialist society for psychosomatic medicine should be the basis of EAPM. Members should inform “physically oriented care givers” in different specialties about the existence, origin, and treatment of psychosomatic disturbances (see above). EAPM members - CL psychiatrists/psychosomatic physicians- are seen as specialists (it remains unclear if for all diagnoses mentioned above in all specialties of bio-psycho-social medicine or only for the limited diagnoses of anxiety, depression, somatoform disorders in C/L-psychiatry or in the psychosomatic specialty). In this sense psychosomatic medicine is not the same as behavioural medicine [8] and the main focus of this society certainly does not represent the “art of healing” applied by all physicians [28].

## Conclusion

We have detected different ways of understanding and interpreting the “medical field”

- The main difference between C-L Psychiatry and Psychosomatics seems to be the point of view: should we consider Psychosomatic Medicine separately as psychosomatic, psychiatric, or psychological experts in the field of medicine and regular care? Or, should we work as primary care physicians observing the interaction with the patient and his or her subjective experiences from their respective fields [4]?
- Translating this view to the scientific concept level: Psychosomatic/behavioural perspective represent causality in a bio psycho social view and the C/L-Psychiatry main point of view is an issue of co-morbidity.
- A third important aspect is the severity of (mental) disease, which leads to different types of intervention procedures: the GP, internal medicine and specialized psychotherapeutic/psychiatric level. All have to be evaluated.
- Physicians responsible for CL-psychiatry tend to focus on severe mental diseases in health care and research. They tend to forget the normality and next to normal variation. Severity of mental disease as well as severity of behavioural or sociological disturbances may influence psychosomatic mechanisms as the origin or course of somatic disorders. There seems to be a tendency to generalize and interpret one’s own clinical view or research interest as the whole field of psychosomatic medicine.
- The competition for power and reputation among psychiatrists, specialty physicians, and among psychologists, psychotherapeutic orientations, and psychopharmacological treatment options, render an open discussion in a network difficult.

In our experience from the last four years of EAPM activities the main topics at conferences (Cambridge, Nuremberg, Lulea) have been health care and C-L Psychiatry. Cooperation with other psychosomatic/behavioural societies, with somatic disciplines — internal medicine, gynaecology, skin disease, etc. - remained small. Within two pre-conferences of the last three meetings, the main psychosomatic cooperation partner was a psychiatrist organization of the APM. In Europe, C/L psychiatrists and some psychosomatic specialists have found a place to meet and discuss issues. Until now there has been limited success in integrating ISBM and ICPM delegates and symposia in EAPM conferences or vice versa.

For the field of psychosomatic medicine as a whole and for its researchers, the situation now is not bad: Psychosomatic/behavioural medicine has reached valuable basic results in a growing field. But, in psychosomatic and behavioural medicine there are competing societies and meetings, thus there is little chance to go to all meetings and it is difficult to choose. It is also a waste of resources. We had hopes for the development of a stimulating and easily accessed website – a sort of Psychosomatic Facebook page, but it took more time than we thought to achieve this. Our expectations to strengthen the psychosomatic movement by his unification have not been realized.

Large research initiatives are difficult to organize successfully. The involvement in large empirical studies has been reduced due to the animosities between the interests of various groups who are dominating and pushing the common interest and importance of progress in knowledge into the background. It is still worthwhile to maintain outstanding standards of psychosomatic research, care, and training in cooperation or competition with other organizations. In summary, we are on the right way, but we have forgotten some aims of the ENPM and we are not sure if EAPM, ICPM, ISBM or other societies involved in psychosomatic medicine are willing to follow. The scene looks very society-focused (EAPM, ICPM, ACPM, APS, ISBM) and does not easily integrate and coordinate research and health care activities in the psychosomatic/behavioural field. But, the ideas of ENPM are still valid.

New knowledge has been reached and therapeutic measures have been able to prolong lives and improve the general health status in certain countries and groups. The connection between mind and brain is being explored. The time has come to implement the spectacular findings of last decades. There is the possibility to communicate through websites and at conferences. Perhaps in the future younger members of these societies will pursue our ideas and proposals within their societies or networks.

## Abbreviations

ACPM: Asian College of Psychosomatic Medicine
APM: American Academy of Psychosomatic Medicine
APS: American Society of Psychosomatic Medicine
C/L: Consultation/Liaison
CBT: Cognitive behavioural therapy
EABCT: European Association for Behavioural and Cognitive Therapies
EAPM: European Association of Psychosomatic Medicine - The European Association of Consultation-Liaison Psychiatry, Psychosomatic Medicine and Integrated Care
ENPM: European Network of Psychosomatic Medicine
ICPM: International College of Psychosomatic Medicine
IJBM: International Journal of Behavioural Medicine
ISBM: International Society of Behavioural Medicine
JPR: Journal of psychosomatic research
Psychother Psychosom: Psychotherapy and psychosomatics
